# Increased peripherin in sympathetic axons innervating plantar metatarsal arteries in STZ-induced type I diabetic rats

**DOI:** 10.3389/fnins.2014.00099

**Published:** 2014-05-07

**Authors:** Niloufer J. Johansen, Tony Frugier, Billie Hunne, James A. Brock

**Affiliations:** Department of Anatomy and Neuroscience, University of MelbourneMelbourne, VIC, Australia

**Keywords:** streptozotocin, type I diabetes, peripherin, β-tubulin III, sympathetic perivascular nerves

## Abstract

A common characteristic of axonopathy is the abnormal accumulation of cytoskeletal proteins. We recently reported that streptozotocin (STZ)-induced type 1 diabetes produced a change in the morphology of sympathetic nerve fibers supplying rat plantar metatarsal arteries (PMAs). Here we investigated whether these morphological changes are associated with axonal accumulation of the type III intermediate filament peripherin and the microtubule protein β-tubulin III, as both are implicated in axonal remodeling. PMAs from hyperglycemic STZ-treated rats receiving a low dose of insulin (STZ-LI) were compared with those from normoglycemic STZ-treated rats receiving a high dose of insulin (STZ-HI) and vehicle-treated controls. Western blotting revealed an increase in protein expression level for peripherin in PMAs from STZ-LI rats but no change in that for β-tubulin III. In addition, there was an increase in the number of peripherin immunoreactive nerve fibers in the perivascular nerve plexus of PMAs from STZ-LI rats. Co-labeling for peripherin and neuropeptide Y (a marker for sympathetic axons) revealed that peripherin immunoreactivity increased in sympathetic axons. None of these changes were detected in PMAs from STZ-HI rats, indicating that increased peripherin in sympathetic axons of STZ-LI rats is likely due to hyperglycemia and provides a marker of diabetes-induced nerve damage.

## Introduction

Recently we demonstrated impaired sympathetic neurovascular transmission in plantar metatarsal arteries (PMAs) from rats with streptozotocin (STZ)-induced diabetes, a well-studied model of type I diabetes (Johansen et al., 2013). In PMAs from STZ-treated rats that received no insulin, the reduction in nerve-evoked vasoconstriction was associated with a decrease in perivascular nerve fiber density. By contrast, in PMAs from hyperglycemic STZ-treated rats that received a low dose of insulin (i.e., a dose insufficient to affect systemic glycemia), neither neurovascular transmission nor perivascular nerve fiber density were changed. However, in both groups of hyperglycemic STZ-treated rats, the PMA perivascular nerve fibers were similarly thickened and had increased tyrosine hydroxylase (TH) immunoreactivity. Therefore, it is possible that diabetes produces changes to the sympathetic innervation of PMAs before functional changes can be detected. For this reason it is important to identify diabetes-induced changes to the perivascular sympathetic nerves that precede disruption of neurovascular function.

In both animals and humans, present evidence indicates that diabetes does not cause significant loss of sympathetic neurons (Schmidt, 2002). Microscopic studies of perivascular axons in mesenteric and tail arteries from rats with STZ-induced diabetes have not revealed any structural changes (Schmidt et al., 1988; Belai et al., 1996; Speirs et al., 2006; Johansen et al., 2013). Therefore, the sympathetic neurons supplying the PMAs appear to be selectively vulnerable to the effects of type 1 diabetes. It has been reported that STZ-induced diabetes produces swelling of sympathetic axons supplying the intestine, corpus cavernosum and pineal gland (Schmidt et al., 1981; Morrison et al., 2007; Tsai et al., 2008). In mesenteric nerves supplying the distal ileum and colon of diabetic rats, Schmidt et al. (Schmidt et al., 1981, 1988; Schmidt and Scharp, 1982; Clark and Schmidt, 1984) have described both degenerating and regenerating sympathetic axons. Indeed the number of axons in the mesenteric nerves increased, suggesting that diabetes stimulates axon sprouting (Schmidt and Scharp, 1982). A increase in sympathetic nerve terminal density has also been reported in the heart of rats with STZ-induced diabetes (Felten et al., 1982).

Therefore, we hypothesized that nerve fiber thickening in perivascular nerve plexus of PMA might be due to both abnormal increases in axon diameter and sprouting of sympathetic axons within the nerve fibers. In sympathetic ganglia, diabetes-induced increases in axon diameter were associated with the accumulation of the type III intermediate filament peripherin (Schmidt et al., 1997), which has recently been demonstrated to be a normal subunit of neurofilaments in peripheral neurons (Yuan et al., 2012). This finding accords with the demonstration that axonal caliber is proportional to the number of neurofilaments (see Holmgren et al., 2012). Therefore, to assess if diabetes might be having a similar effect on sympathetic nerves supplying PMAs, we assessed if there were increases in immunolabeling for peripherin. In addition, we assessed if diabetes increased immunolabeling for the neuron specific neurotubule protein β-tubulin III, which is an axon growth associated tubulin that is upregulated during regeneration of peripheral axons and incorporated into the neurotubules in newly grown axons (Moskowitz and Oblinger, 1995; Hoffman, 2010). This pan axonal marker also allowed us to assess changes to the entire perivascular nerve plexus. We also used Western blots to assess if diabetes increased protein expression levels for peripherin and β-tubulin III in the whole tissue.

## Materials and methods

### Animal handling and tissues

All experiments were approved by the University of Melbourne Animal Ethics Committee and conformed to the Australian code of practice for the care and use of animals for scientific purposes. Male Wistar rats, 8–10 weeks old, from the Animal Resources Centre (Canning Vale, WA, Australia), were treated intraperitoneally with 60 mg/kg STZ (Sigma-Aldrich Pty. Ltd., Sydney, NSW, Australia) dissolved in 50 mM citrate buffer (pH 4.5). Age-matched vehicle control animals were treated with an equivalent volume of citrate buffer. Blood glucose levels were measured 5–7 days post-injection using a glucose meter (Accu-Check Performa, Roche Diagnostics Australia Pty Ltd) to confirm that the STZ-treated rats were diabetic (>15 mM glucose). The STZ-treated rats were then divided into two groups, one of which received a low dose of insulin and were hyperglycemic (STZ-LI; blood glucose >15 mM) and the other received a high dose of insulin and were normoglycemic (STZ-HI; blood glucose <15 mM). Insulin was delivered by sustained-release implants (Linplant, Linshin Canada Inc.) inserted subcutaneously beneath the nuchal skin. The STZ-LI rats received half a Linplant pellet (~1 insulin unit/day) at 1 week and again at 6–7 weeks post-STZ treatment. The first cohort of STZ-HI rats received 2 Linplant pellets (~4 insulin units/day) at 1 week and 6–7 weeks after STZ treatment. To better maintain normoglycemic levels and prevent the animals from becoming transiently hypoglycemic, the second cohort of STZ-HI rats initially received 1.5 pellets at 1 week post-STZ treatment and then received additional pellets (1–1.5 pellets) at ~30 day intervals when the blood glucose levels increased to between 10 and 15 mM. Blood glucose levels and body weights were monitored at weekly intervals until rats were terminated 12–13 weeks after the induction of diabetes. Animals were maintained on a 12:12 h light-dark cycle and were provided with food and water *ad libitum*.

At termination the animals were deeply anesthetized with isoflurane and exsanguinated by cutting the carotid arteries. Approximately 1 ml of blood was collected in EDTA tubes from all rats to determine the glycosylated hemoglobin levels by high-performance liquid chromatography (CLC330 GHb Analyzer, Primus, Kansas City, MO). From each hind paw, the median plantar artery and the five PMAs branching from the plantar arch were dissected in cold phosphate buffered saline (PBS) containing 1:1000 of 50 mM protease inhibitor phenylmethylsulfonyl fluoride (PMSF; Sigma-Aldrich). The first two PMAs from the medial side of each hind paw were used for immunohistochemistry. The remaining arteries from both hind paws were pooled for each animal and snap frozen in liquid nitrogen and stored at −80°C.

### Tissue sample preparation for western blots

The following solutions were added to each arterial tissue sample (wet weight ≤30 mg): 300 μl RIPA buffer (Sigma-Aldrich), 3 μl protease inhibitor cocktail (Sigma-Aldrich), 0.5 μl of 50 mM PMSF. Samples were kept at 4°C during the following procedures. Arteries were diced using scissors, sonicated for 3–4 s twice (speed 6; XL2000, Misonix, NY, USA), centrifuged at ~18,000 g for 5 min, and then the supernatant was collected. As per manufacturer's instructions, the protein content of each sample was quantified using Bio-Rad's Dc™ Protein Assay Kit (Bio-Rad Laboratories Pty., Ltd., Gladesville, NSW, Australia). Samples were aliquoted in volumes required to load 20 μg of protein per well on the gels and stored at −80°C.

### Western blots

Proteins were separated using a Mini-PROTEAN 3 Electrophoresis System (Bio-Rad). To each sample, an equal volume of Laemmli sample buffer (Bio-Rad) and 2 μL of 2-mercaptoethanol (Sigma-Aldrich) per 50 μL of sample was added. Samples were heated to ~100°C for 5 min and loaded into the wells of Mini-PROTEAN® TGX™ precast 4–20% gradient gels (Bio-Rad) immersed in Tris/glycine buffer (2.5 mM Tris, 19.2 mM glycine, pH 8.3). One well in each gel was loaded with 6 μl of molecular weight marker (Bio-Rad). To allow comparisons between the protein content of samples run on different gels, at least one well per gel contained a protein sample common to all gels (internal standard). Proteins were separated electrophoretically at 100 V for 100 min at room temperature. After separation, the proteins were transferred to nitrocellulose membranes (pore size 0.22 μm; LI-COR, NE, USA) using a Mini Trans-Blot® Electrophoretic Transfer Cell (Bio-Rad). Each tank was filled with Tris/glycine buffer containing 20% (v/v) methanol and transfers were carried out overnight at 30 V and 4°C. After staining with ponceau red (Sigma-Aldrich) to confirm protein transfer was successful, the membranes were washed 3× with distilled water and air dried for 1 h at room temperature.

### Antibody labeling for western blots

Membranes were blocked in casein blocking buffer (LI-COR, NE, USA) for 1 h at room temperature. Both, primary and secondary antibodies were diluted in casein buffer containing 0.01% Tween-20 (Sigma-Aldrich). The membranes were incubated with mouse anti-β-tubulin III (1:1000; Cat. No. MMS-435P, Covance, Princeton, NJ, USA) or rabbit anti-peripherin (1:5000; Cat. No. AB1530, Chemicon-Millipore-Merck, Kilsyth, VIC, Australia) at 4°C overnight. Unbound antibodies were removed by washing with PBS containing 0.001% Tween-20 (3 × 10 min). Membranes immunolabeled for peripherin were then incubated with fluorescent secondary antibody, IRDye® donkey anti-rabbit IgG 800CW (1:1000; LI-COR) for 1 h at room temperature before they were again washed with PBS-0.001% Tween-20 (3 × 10 min) and then with PBS alone (1 × 10 min). β-tubulin III labeling was visualized by applying a biotin-streptavidin step to enhance signal detection. Biotinylated horse anti-mouse IgG antibody (1:5000; Cat. No. BA-2000, Vector Labs, Burlingame, CA, USA) was applied for 1 h at room temperature and then the membrane was washed with PBS-0.001% Tween-20 (3 × 10 min). The membranes were then fluorescently labeled with streptavidin 680LT dye (1:20,000, LI-COR) for 1 h at room temperature before being washed off with PBS-0.001% Tween20 (3 × 10 min) and then with PBS alone (1 × 10 min).

Membranes were imaged using LI-COR's Odyssey® near infrared imager and images were acquired using the program Odyssey v2.1. After the protein of interest was imaged, the membranes labeled with mouse anti-β-tubulin III or rabbit anti-peripherin antibodies were co-labeled with antibodies against β-actin raised in either rabbit (1:1000; Cat. No. 926-42210, LI-COR) or mouse (1:10,000; Cat. No. 926-42212, LI-COR), respectively. Membrane-bound β-actin antibodies were labeled with fluorescent IRDye® donkey anti-rabbit IgG 800CW (1:1000; Cat. No. 926-32213, LI-COR) or IRDye® donkey anti-mouse IgG 680LT (1:5000; Cat. No. 926-68022, LI-COR). After β-actin labeling, both the protein of interest and housekeeper protein were imaged simultaneously.

The integrated fluorescence intensity of the bands was measured using ImageJ (NIH, Bethesda, USA). Expression level for the protein of interest (i.e., β-tubulin III or peripherin) in each sample was determined relative to that of β-actin. As reported in the results, we confirmed that the protein content of the β-actin bands did not differ among the samples from the control, STZ-HI and STZ-LI groups of rats. To correct for inter-gel variability, the relative values for the test samples on each gel were normalized to that for the internal standard on the same gel.

### Tissue fixation and immunohistochemistry

PMAs were fixed at their *in vivo* length in Zamboni's fixative overnight at 4°C. Samples were washed with dimethyl sulfoxide (3 × 10 min) followed by PBS (3 × 10 min), and stored in PBS containing 0.01% (w/v) sodium azide at 4°C. In all immunohistochemical experiments, PMAs collected from STZ-treated and control rats terminated on the same day were performed in parallel on the same slide (i.e., for each set of immunolabels). Tissues were blocked for 1 h in PBS containing1% (v/v) Triton (Sigma-Aldrich) and 10% (v/v) normal horse serum. Tissues were labeled in different combinations (presented in the results) with the following primary antibodies: rabbit anti-peripherin (1:500), mouse anti-β-tubulin III (1:750), sheep anti-neuropeptide Y (NPY; 1:2000; Clone E2210, Gift from J. Oliver) and rabbit anti-calcitonin gene-related peptide (CGRP; 1:1000; Cat. No. T-4032, Bachem, Bubendorf, Switzerland). Primary antibodies were visualized using fluorescent secondary antibodies raised in donkey (Molecular Probes, Inc., OR, USA); anti-rabbit 488 (1:1000; Cat. No. A21206), anti-mouse 647 (1:500; Cat. No. A31571), and/or anti-sheep 647 (1:500; Cat. No. A21448). No primary antibody controls were performed for each sample to ensure specificity of secondary antibodies. Antibodies were diluted in antibody diluent (290.9 mM NaCl, 7.5 mM Na_2_HPO_4_, 2.5 mM NaH_2_PO_4_, and 0.1% (w/v) sodium azide; pH 7.1). Tissues were incubated with the primary antibody overnight at 4°C. Excess antibody was washed off with PBS (3 × 10 min) and secondary antibodies were applied for 1 h at room temperature. Samples were then washed with PBS (3 × 10 min) and mounted and coverslipped on Starfrost® slides (Waldemar Knittel Glasbearbeitungs GmbH, Braunschweig, Germany) with fluorescence mounting medium (Dako North America, Inc., CA, USA). Samples were viewed with a Zeiss Pascal confocal microscope system and imaged as Z-stacks through the entire adventitial thickness.

### Data analysis

All statistical analyses were performed using SPSS 19 (IBM Corp, Armonk, NY, USA) or GraphPad Prism (GraphPad Inc., La Jolla, CA, USA). For Western blotting experiments and the measurements of net body weight gain, % glycosylated hemoglobin and blood glucose levels at termination, comparisons were first made among control, STZ-HI and STZ-LI groups using One-Way ANOVA or a Kruskal–Wallis test if the data variance differed significantly among the experimental groups (assessed using Levene's test). *Post-hoc* pairwise comparisons were made with Tukey's honest significant difference (HSD) tests or Dunn's tests. When parametric tests were used the data is expressed as means and standard errors of the mean (SEM), whereas when non-parametric tests were used the data are presented as medians and interquartile ranges.

The β-tubulin III and peripherin immunolabeled perivascular axon plexus innervating PMAs was quantified using maximum intensity Z-projection images collected with a ×63 objective (512 × 512 pixels, pixel size 0.24 μm^2^). This was done by collecting the pixel intensity values along two horizontal lines placed at the same points on all images (one in the upper half and the other in the lower half of the image) using the line profile function in ImageJ (National Institute of Health, Bethesda, MD) (see Figure 2 for representative line profiles). After subtracting the mean maximum background pixel intensity value (determined from 10 points on the image where there was no detectable fluorescence), the number and widths of the nerve fiber intercepts along the line profiles (i.e., regions with pixel values >0) were determined using an in-house routine composed in Igor Pro (Wavemetrics, Lake Oswego, OR, USA). ImageJ was used to measure the percentage area of the vessel surface covered by the β-tubulin III or peripherin immunoreactive (IR) nerve plexus and the integrated peripherin and β-tubulin III-IR fluorescence per 100 μm^2^ of vessel surface (for both of these measures the mean maximum background pixel intensity value was used as the threshold for detecting nerve fibers). For each nerve plexus measure, one-sample Student's *t*-tests were used to determine if the measures in arteries from STZ-treated animals expressed as a percentage of those in the control tissues collected on the same day (and processed in parallel) differed significantly from 100%. For each animal, the measures were the average obtained from at least three image Z-stacks.

For studies assessing co-localization of peripherin-IR and NPY-IR, horizontal pixel intensity line profiles for each label were obtained at the same two points on each image (one in the upper half and the other in the lower half of the image) as described above (see Figure 6 for representative line profiles). Prior to assessing co-localization, the mean maximum background pixel intensity value for each label (determined as described above) was subtracted from the line profile for that label. For each pair of line profiles collected at the same point on the image, co-labeling was assessed by determining the percentage of fluorescence peaks along each line profile at which the pixel value at the same location on the other line profile was >0. This allowed us to determine the percentage of peripherin-IR intercepts that were also NPY-IR, and vice versa. Additionally, the integrated NPY-IR fluorescence per 100 μm^2^ of vessel surface was measured (as described above).

## Results

### Animals

**Figure 1:**
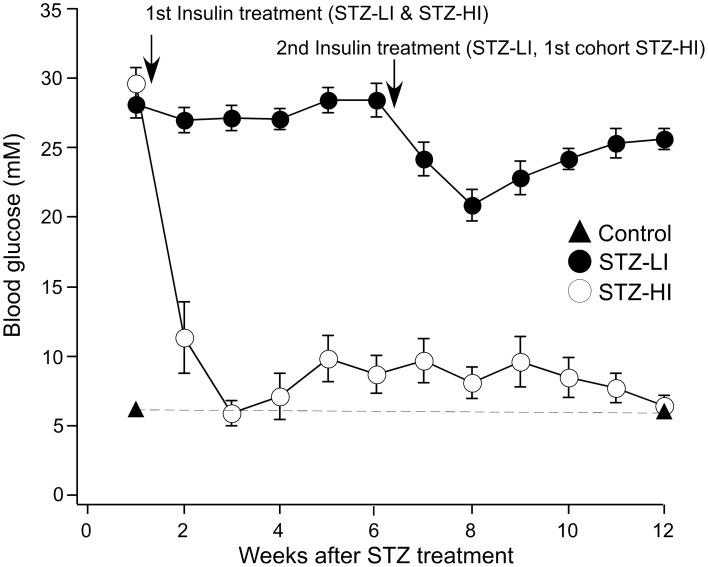
**Blood glucose levels (mM) measured at weekly intervals after i.p. injection of streptozotocin (STZ)**. Before the insertion of insulin pellets (at 1 week) all STZ-treated rats had elevated blood glucose levels. Throughout the 12-week period, STZ-treated rats given a low dose of insulin (STZ-LI, *n* = 22) maintained blood glucose levels >15 mM (i.e., hyperglycemic). Those given a high dose of insulin (STZ-HI, *n* = 19), at 3 weeks and thereafter had blood glucose measurements <15 mM. The graph also shows blood glucose measurements at 1 week and at 12 weeks in citrate buffer-treated control rats (*n* = 30) and these data points are joined by the dashed line. The first arrow indicates the time at which the STZ-LI and STZ-HI rats received their first insulin pellet(s). The second arrow indicates the time the STZ-LI rats and the first cohort of STZ-HI rats received their second insulin pellet(s). The other STZ-HI rats were given additional insulin pellets when their blood glucose increased to between 10 and 15 mM.

All STZ-treated rats had elevated blood glucose levels (>20 mM) prior to insulin treatment (at 1 week after STZ treatment), and the blood glucose levels for the STZ-LI rats was >15 mM throughout the 12 week period (Figure 1). In comparison, the blood glucose levels of STZ-HI rats were greatly reduced by insulin treatment (Figure 1; between STZ-LI and STZ-HI group comparison *P* < 0.001). At termination, the blood glucose levels did not differ between control and STZ-HI rats, but both had levels that were significantly lower than those for STZ-LI rats (Table 1). The net body weight gain for STZ-LI animals at termination was less than for both STZ-HI and control rats, and that for the STZ-HI rats was also less than that for the controls (Table 1). Terminal % glycosylated hemoglobin levels in blood from STZ-LI rats (~16%) were about twice those in STZ-HI rats (~8%), but levels in STZ-HI rats were slightly higher than those in control rats (~5%) (Table 1).

**Table 1:** **Net body weight gains, % glycosylated hemoglobin levels and blood glucose levels at termination in streptozotocin (STZ)-treated rats receiving a high (STZ-HI) or low (STZ-HI) dose of insulin and their controls**.

**Groups**		**Net body weight gain (g)**	**% Glycosylated hemoglobin**	**Terminal blood glucose level (mM)**
Control (*n* = 30)		241 ± 9	5.1 ± 0.4	6.0 (5.4–6.7)
STZ-HI (*n* = 19)		196 ± 9	7.7 ± 0.7	5.8 (4.7–8.5)
STZ-LI (*n* = 22)		104 ± 8	16.4 ± 0.5	26.1 (23.8–27.8)
Comparison between groups		*P* < 0.001 *F* = 63.55	*P* < 0.001 *F* = 136.0	*P* < 0.001 Kruskal-Wallis statistic = 45.05
Pairwise comparison	Control vs. STZ-HI	*P* < 0.001	*P* < 0.001	*P* = 0.61
	Control vs. STZ-LI	*P* < 0.001	*P* < 0.001	*P* < 0.001
	STZ-HI vs. STZ-LI	*P* < 0.001	*P* < 0.001	*P* < 0.001

Data are presented as means and s.e.m.'s or medians and interquartile ranges (in parentheses). Comparisons between the control, STZ-HI, and STZ-LI groups were initially made using One-Way ANOVA or the Kruskal-Wallis test and pairwise comparisons between the groups were made with Tukey's HSD or Dunn's tests.

### A subset of the perivascular axons innervating the PMAs was peripherin-IR

**Figure 2:**
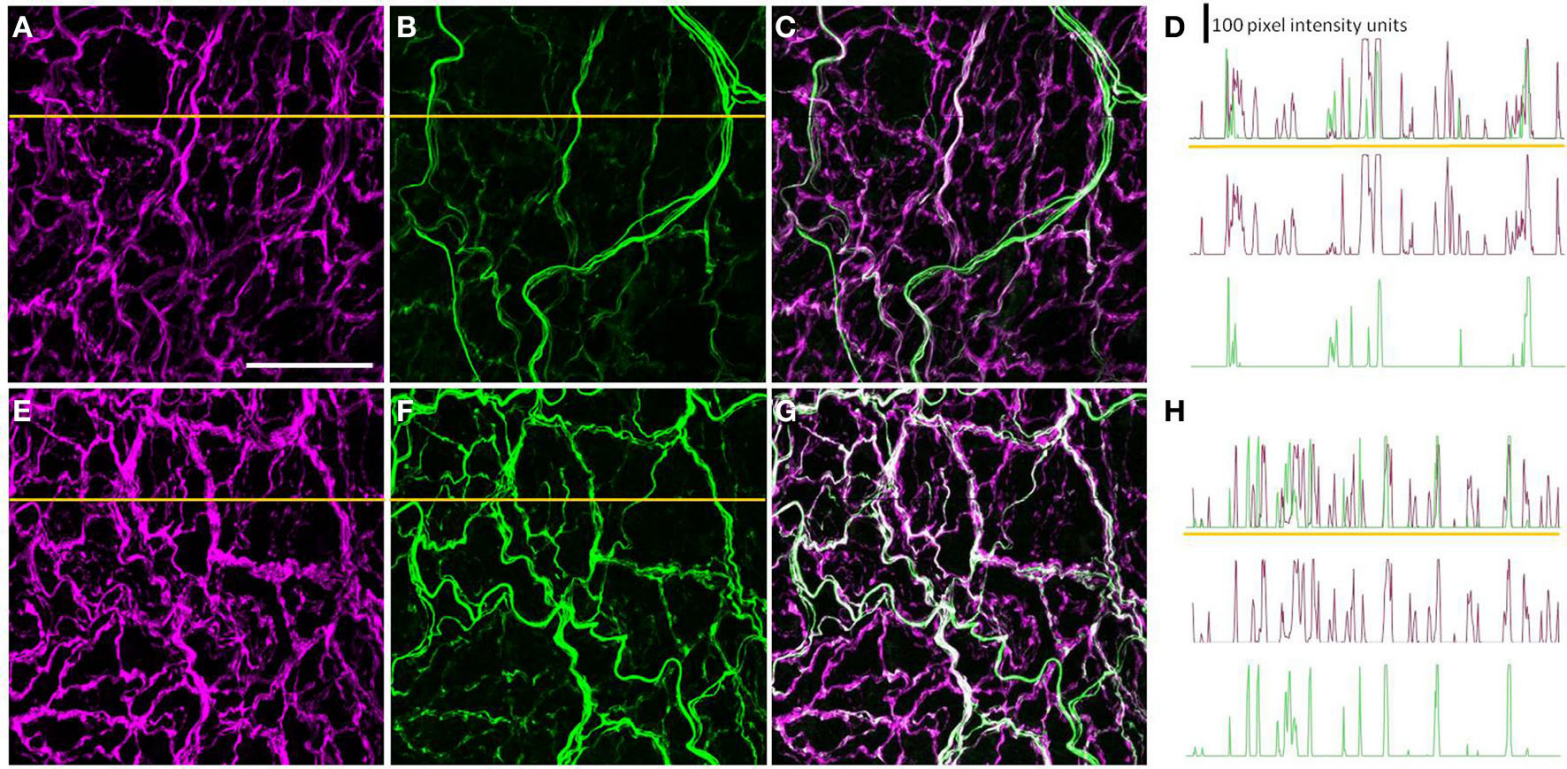
**Images showing immunolabeling for β-tubulin III (magenta; A,E) and peripherin (green; B,F) and the combination of both proteins (C,G) in the perivascular nerve plexus of a plantar metatarsal artery (PMA) from a control rat (A–C) and a streptozotocin (STZ)-treated rat (E–G) that received a low dose of insulin (STZ-LI)**. In comparison with the PMA from the control rat, the β-tubulin III-immunoreactive (IR) nerve fibers were thickened in the PMA from the STZ-LI rat. In addition, there was an increase in the number of fine peripherin-IR fibers in the PMA from the STZ-LI rat. **(D,H)** show representative pixel intensity line profiles (only pixel intensity values above the mean maximum background level are shown) for β-tubulin III-IR (magenta) and peripherin-IR (green) collected along the orange lines indicated on **(A,E)** and **(B,F)**, respectively (the pixel intensity scale bar in **D** also applies in **H**). The images were collected with a ×63 objective and the length scale bar in **(A)** indicates 50 μm and applies to all images and line profiles.

Most, if not all, of the axons within the nerve plexus of PMAs are β tubulin III-IR (Johansen et al., 2013). Figure 2 shows images for PMAs from a control (Figures 2A–C) and a STZ-LI rat (Figures 2E–G) immunolabeled for both β-tubulin III and peripherin. Only the thicker β-tubulin III-IR nerve fibers were strongly peripherin-IR in PMAs from control and STZ-HI rats (not shown). By contrast, in the PMAs from the STZ-LI rats, there was an increase in peripherin-IR in the finer nerve fibers (c.f. Figures 2B,F). Figure 2 also shows representative line profiles used for quantifying the β-tubulin III-IR and peripherin-IR nerve plexus (see below) collected from the images (Figures 2D,H).

### PMA β-actin protein expression levels did not vary among treatment groups

**Figure 3:**
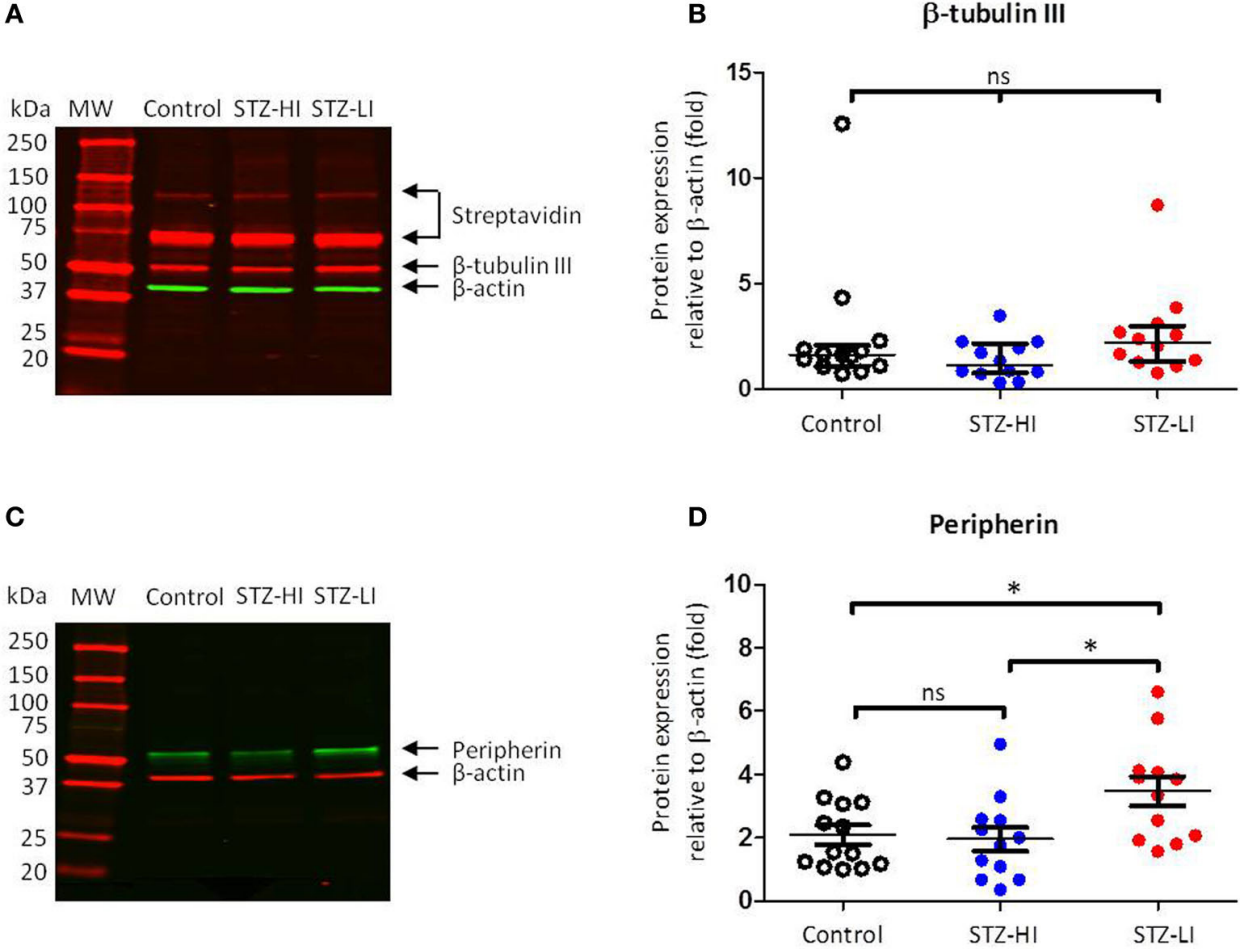
**β-tubulin III and peripherin protein levels in plantar metatarsal arteries (PMAs) from control rats (*n* = 13) and streptozotocin (STZ)-treated rats that received a low (STZ-LI, *n* = 12) or a high (STZ-HI, *n* = 12) dose of insulin. (A,C)** Representative Western blots for β-tubulin III **(A)** and peripherin **(C)** in PMAs from a control, a STZ-HI and a STZ-LI rat. The predicted molecular weights (MW) for β-tubulin III, peripherin and β-actin were approximately 50, 57, and 45 kDa, respectively. **(B,D)** Protein expression for β-tubulin III **(B)** and peripherin **(D)** was measured relative to loading control β-actin. In **(B)**, the median and interquartile range are indicated whereas in **(D)** the mean and s.e.m. are indicated. There were no detectable differences in β-tubulin III protein expression levels between PMAs from control, STZ-HI, and STZ-LI rats. Peripherin protein expression in PMAs from STZ-LI rats was greater than that in PMAs from control and STZ-HI rats. Initial comparisons between the groups were made using a One-Way ANOVA (in **D**) or the Kruskal-Wallis test (in **B**). In **(D)**, pairwise comparisons between the groups were made with Tukey's tests (ns = not significant; ^*^P < 0.05).

The β-actin protein expression in all PMA samples was identified as a 45 kDa band (Figures 3A,C). The intensity values relative to the internal standard for β-actin protein expression for arteries from each treatment group (i.e., controls, STZ-HI and STZ-LI) were compared to ensure diabetes did not alter β-actin protein content. Among the three groups, no differences in β-actin protein expression (Table 2) were detected. β-actin was therefore an appropriate loading control for standardizing β-tubulin III and peripherin protein expression for PMAs.

**Table 2:** **Comparison of β-actin protein expression levels (relative to the internal standard) in arterial samples from control rats and streptozotocin (STZ)-treated rats receiving a high (STZ-HI) or low dose of insulin (STZ-LI)**.

**Control**	**STZ-HI**	**STZ-LI**
**PLANTAR METATARSAL ARTERIES**		
2.64	1.56	2.09
(1.51–3.70)	(1.19–2.83)	(1.77–5.23)
*n* = 13	*n* = 12	*n* = 12
Kruskal-Wallis Test *P* = 0.22		
Kruskal-Wallis statistic = 3.07		

Data are presented as medians and interquartile ranges (in parentheses). Statistical comparisons between the control, STZ-HI, and STZ-LI groups were made using the Kruskal Wallis test.

### β-tubulin III content in PMAs was not changed by diabetes

Three bands were identified by streptavidin detection on the Western blots for β-tubulin III (Figure 3A). The first two bands (from the top) had much higher molecular weights (~120 and ~75 kDa) than that expected for β-tubulin III (~50 kDa). These bands were also present on the membranes where no primary antibody or biotinylated antibody had been applied and are therefore most likely due to streptavidin binding to endogenous biotin or a biotin-like protein present in the samples (McKay et al., 2008). The third band at ~50 kDa was consistent with the predicted molecular weight of β-tubulin III (Figure 3A). There was no difference in β-tubulin III content between PMAs from control, STZ-HI and STZ-LI rats (Kruskal–Wallis test, *df* = 34, *P* = 0.14; Figure 3B).

### The thickness of β-tubulin III immunoreactive nerve fibers and the area of the vessel surface covered by these fibers were increased in PMAs from STZ-LI rats

**Figure 4:**
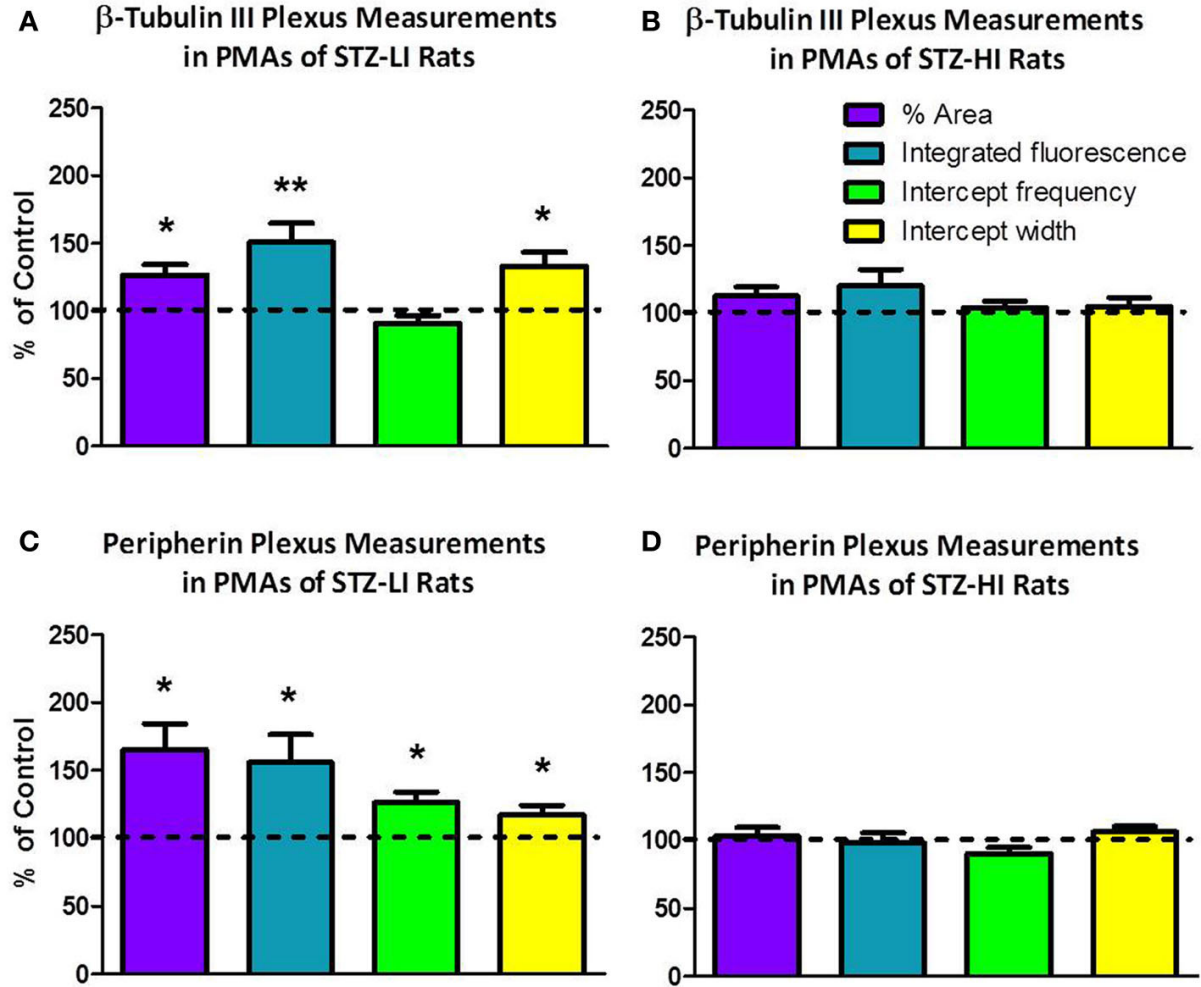
**The percent area of the vessel surface covered by the immunolabeled nerve plexus (% area), integrated fluorescence/100 μ m^2^ of vessel surface (integrated fluorescence), and the frequency and width of intercepts along line profiles of plantar metatarsal arteries (PMAs) immunolabeled for β-tubulin III (A,B) and peripherin (C,D) from streptozotocin (STZ)-treated rats that received a low (STZ-LI; A,C) or a high (STZ-HI; B,D) dose of insulin**. All these measures are expressed as a percentage of those in paired control tissues (i.e., terminated on the same day). Relative to the controls, the β-tubulin III-immunoreactive (IR) and peripherin-IR nerve plexus of PMAs from STZ-LI rats (*n* = 8) had increases in the % area, the integrated fluorescence and the widths of the intercepts along the line profiles. In addition, in PMAs from STZ-LI rats there was an increase in the frequency of peripherin-IR intercepts along the line profiles. For PMAs from STZ-HI rats (*n* = 9), none of the measures of the β-tubulin III-IR or peripherin-IR nerve plexus differed from those of their paired controls. The data are presented as mean and s.e.m. Statistical comparisons were made with one-sample *t*-tests (^*^P < 0.05, ^**^P < 0.01).

The percentage area of the vessel surface covered by the β-tubulin III-IR nerve plexus and the integrated β-tubulin III-IR immunofluorescence per 100 μm^2^ of the vessel surface were significantly larger (~20 and ~45%, respectively) in PMAs from STZ-LI rats compared to their controls (Figure 4A). In PMAs from STZ-LI rats, the frequency of the β-tubulin III-IR intercepts along the line profiles did not differ significantly from those of their controls but the widths of the intercepts were ~30% larger (Figure 4A). For the PMAs from STZ-HI animals, all measures of the β-tubulin III-IR nerve plexus did not differ from those of their controls (Figure 4B).

### Peripherin content was significantly increased in PMAs from STZ-LI rats

Western blots for peripherin had a single protein band located at a position slightly greater than 50 kDa (Figure 3C) and this is consistent with the predicted molecular weight for peripherin (57 kDa). There was a significant difference in the peripherin content among PMAs from control, STZ-HI, and STZ-LI rats (Figure 3D; One-Way ANOVA; *F* = 4.73, *df* = 34, *P* = 0.02). *Post-hoc* comparisons using Tukey's tests revealed that the peripherin protein expression levels were increased in PMAs from STZ-LI rats compared to both control (*P* = 0.04) and STZ-HI (*P* = 0.02) rats. There was no significant difference between the peripherin content of PMAs from control and STZ-HI rats (*P* = 0.96).

### The density of peripherin-IR nerve fibers was increased in PMAs from STZ-LI rats

The percentage area of the vessel surface covered by the peripherin-IR nerve plexus and the integrated peripherin-IR fluorescence per 100 μm^2^ of the vessel surface were both increased by ~60% in PMAs from STZ-LI rats compared to their controls (Figure 4C). Additionally, compared to PMAs from control rats, there was an approximately 25% increase in the frequency of peripherin-IR intercepts along the line profiles in PMAs from STZ-LI rats (Figure 4C). The widths of the intercepts along the line profiles were also increased (~15%) in PMAs from STZ-LI rats (Figure 4C). For PMAs from STZ-HI animals, all measures of the peripherin-IR nerve plexus did not differ from those of their controls (Figure 4D).

### Labeling for peripherin was increased in nerve fibers that were co-labeled for NPY

**Figure 5:**
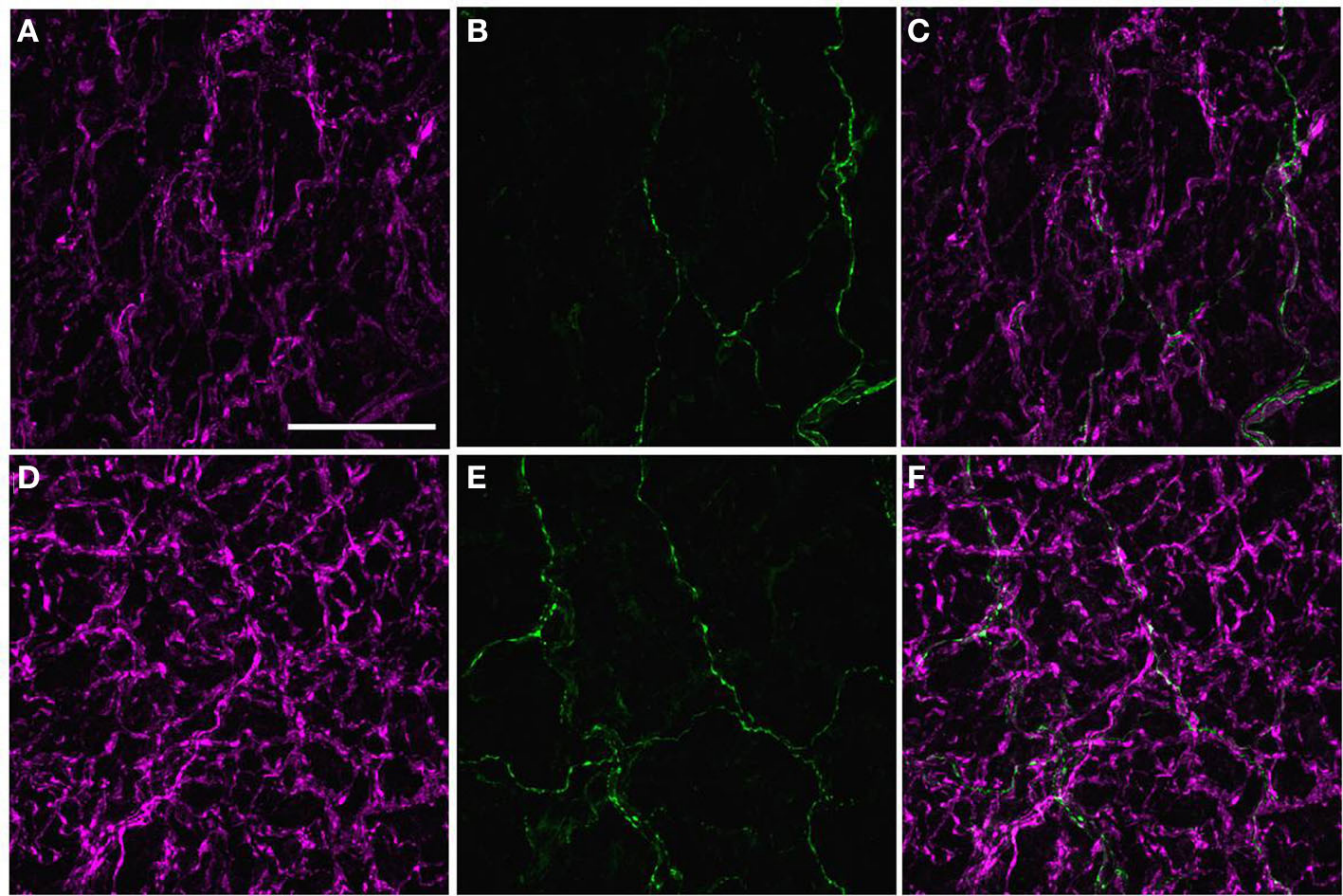
**Images displaying immunoreactivity (IR) for neuropeptide Y (NPY)(magenta; A,D) and calcitonin gene-related peptide (CGRP) (green; B,E) and the combination of both markers (C,F) in the perivascular nerve plexus of a plantar metatarsal artery (PMA) from a control rat (A–C) and a streptozotocin (STZ)-treated rat (D–F) that received a low dose of insulin (STZ-LI)**. In both vessels, the majority of nerve fibers in the perivascular nerve plexus were NPY-IR and only a few axons were CGRP-IR. In comparison with the PMA from a control rat, immunofluorescence for NPY was increased in the PMA from the STZ-LI rat. The images were collected with a ×63 objective and the scale bar in **(A)** indicates 50 μm and applies to all images.

The majority of axons within the perivascular nerve plexus of PMAs were NPY-IR and, relative to controls (Figure 5A), there was increased labeling for NPY-IR in the nerve fibers supplying PMAs from STZ-LI rats (Figure 5D; % increase in integrated NPY-IR 189 ± 35%. *n* = 8, one sample Student's *t*-test *P* < 0.05). These NPY-IR fibers are almost certainly sympathetic nerve fibers, as we have previously reported that the perivascular nerve plexus of PMAs is comprised primarily of tyrosine-hydroxylase (TH)-IR fibers, with only a small number of peptidergic primary afferent nerve fibers containing CGRP (Johansen et al., 2013). Sciatic nerve injury (Wakisaka et al., 1991, 1992; Ohara et al., 1994) has been reported to increase expression of NPY in the cell bodies of sensory neurons in dorsal root ganglia (DRG). The possibility that diabetes increased NPY expression in the CGRP-IR perivascular nerve fibers can be excluded, as the CGRP-IR fibers did not co-label for NPY-IR in PMAs from either control or STZ-LI rats (Figures 5A–F). In addition, we have previously demonstrated that the density of CGRP-IR fibers in the perivascular nerve plexus is not different between PMAs from control and STZ-LI rats (Johansen et al., 2013). Importantly, diabetes increases labeling for TH-IR in the nerve plexus of PMAs (Johansen et al., 2013). Therefore, increased labeling for both NPY-IR and TH-IR in PMAs from STZ-LI rats may be associated with the thickening of the perivascular sympathetic nerve fibers observed in these vessels.

**Figure 6:**
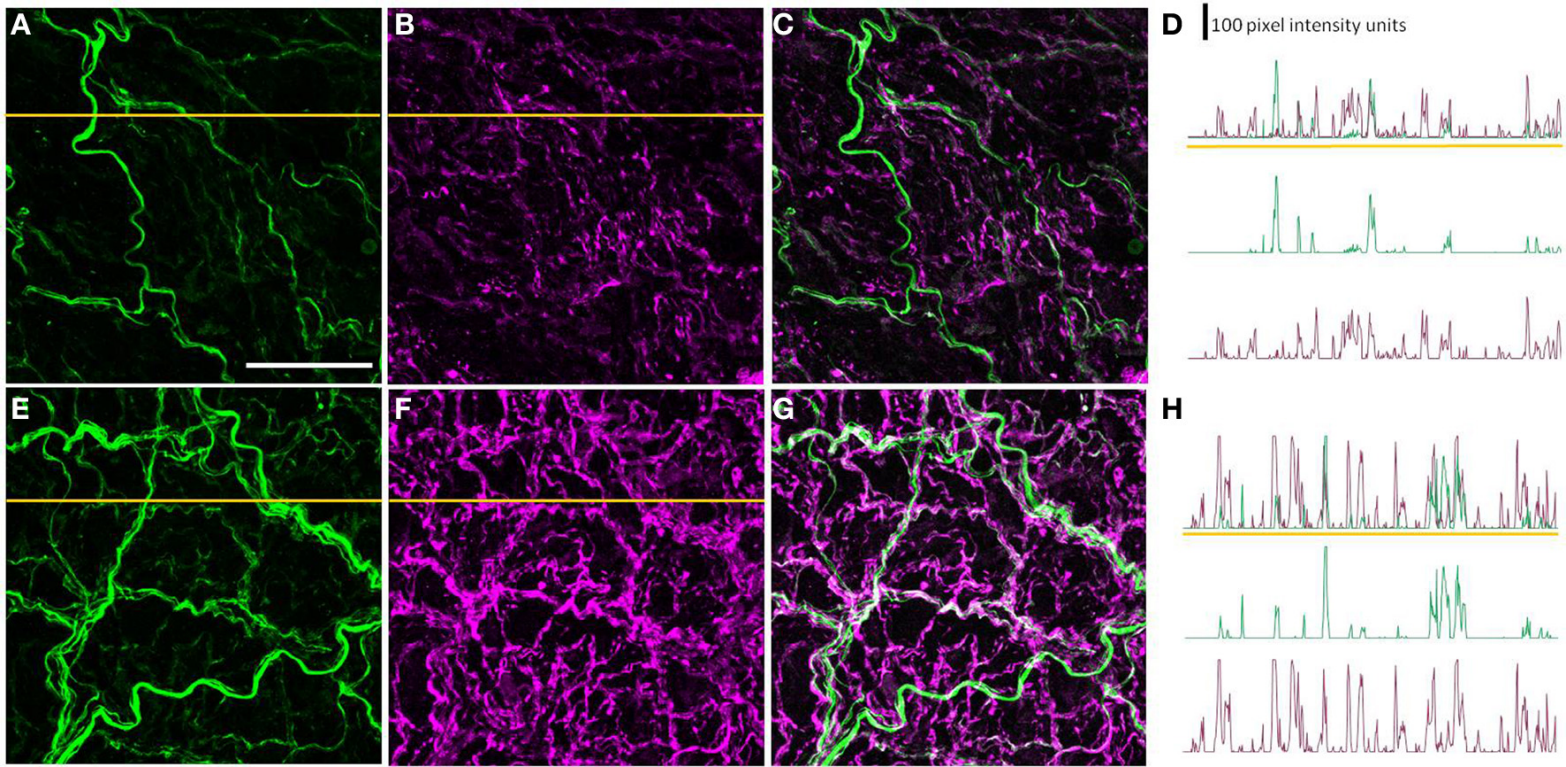
**Images showing immunolabeling for peripherin (green; A,E) and neuropeptide Y (NPY: magenta; B,F) and the combination of both molecules (C,G) in the perivascular nerve plexus of a plantar metatarsal artery (PMA) from a control rat (A–C) and a streptozotocin (STZ)-treated rat (E–G) that received a low dose of insulin (STZ-LI)**. In comparison with the PMA from a control rat, the number of peripherin-IR fibers that were NPY-IR increased in the PMA from the STZ-LI rat. In addition, there was an increase in the thickness and immunofluorescence of the NPY-IR fibers in the PMA from the STZ-LI rat. **(D)** and **(H)** show representative line pixel intensity profiles (only pixel intensity values above the mean maximum background level are shown) for peripherin-IR (green) and NPY-IR (magenta) collected along the orange lines indicated on **(A,E)** and **(B,F)**, respectively (the pixel intensity scale bar in **D** also applies in **H**). The images were collected with a ×63 objective and the length scale bar in **(A)** indicates 50 μm and applies to all images and line profiles.

**Figure 7:**
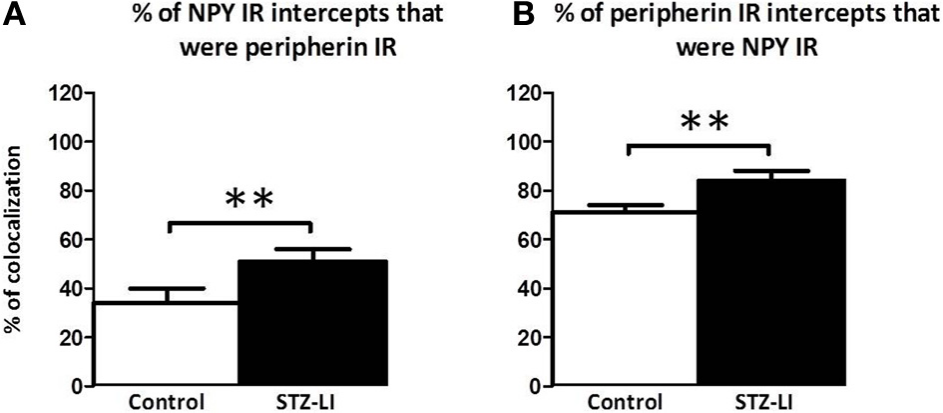
**Percentage of nerve fibers that were co-labeled for peripherin and neuropeptide Y (NPY) in the perivascular nerve plexus of plantar metatarsal arteries (PMAs) from control rats (*n* = 8) and streptozotocin (STZ)-treated rats that received a low dose of insulin (STZ-LI, *n* = 8)**. In comparison with PMAs from control rats, those from STZ-LI rats had an increase in the number of NPY-immunoreactive (IR) intercepts along the line profiles that were peripherin-IR **(A)** and vice versa **(B)**. The data are presented as mean and s.e.m. Statistical comparisons were made with unpaired *t*-tests (^**^P < 0.01).

To determine whether the diabetes-induced increase in peripherin-IR occurred in sympathetic nerve terminals, line profiles were used to determine the percentage of peripherin-IR intercepts that were also NPY-IR (a sympathetic nerve marker), and vice versa, in PMAs from control and STZ-LI rats. Figure 6 shows images for PMAs from a control (Figures 6A–C) and a STZ-LI rat (Figures 6E–G) immunolabeled for both peripherin and NPY, and representative line profiles for peripherin-IR and NPY-IR collected from these images (Figures 6D,H). In control PMAs, the larger diameter fibers that were peripherin-IR showed little or no NPY-IR (the green fibers in the merged image; Figure 6C). In comparison with controls, STZ-LI PMAs had an increase in the number of fibers that were co-labeled for peripherin and NPY (white fibers in the merged image; Figure 6G); expression of NPY and peripherin increased in the thicker and finer nerve fibers, respectively (Figures 6E–G). In PMAs from STZ-LI rats, the percentage of intercepts along the line profiles for NPY-IR that were co-labeled for peripherin-IR was increased compared to that in PMAs from control rats (Figure 7A). Similarly, the percentage of peripherin-IR intercepts that were NPY-IR increased in these vessels (Figure 7B). These findings indicate that peripherin protein expression increased in NPY expressing (i.e., sympathetic) nerve fibers.

## Discussion

This study shows that STZ-induced type I diabetes increased both the thickness of perivascular nerve fibers and the expression of peripherin in the perivascular nerve plexus of PMAs. As the increase in peripherin immunolabeling occurred in nerve fibers that were also NPY-IR, they are sympathetic nerve terminals. As no changes in structure of the perivascular nerve plexus or in peripherin protein expression or immunolabeling were observed in PMAs from STZ-HI rats, the effects observed in PMAs from STZ-LI rats were likely due to hyperglycemia and/or to the reduced neurotrophic action of insulin (Sima, 2008). While there was an increase in the integrated immunofluorescence for β-tubulin III in the perivascular plexus of PMAs from STZ-LI rats, the expression levels for this protein assessed using Western blots were not significantly changed by diabetes. As there was no increase in nerve fiber intercept frequency along the line profiles for β-tubulin III-IR (i.e., nerve fiber density) but the % area of the vessel surface covered by the β-tubulin III-IR nerve plexus increased in PMAs from STZ-LI rats (Figure 4), the increase in integrated immunofluorescence is most readily explained by the increase in neural tissue associated with nerve fiber thickening rather than an increase in the relative expression level of β-tubulin III in the whole tissue (i.e., measured relative to the total tissue β-actin).

Peripherin is a type III intermediate filament protein that is expressed in subpopulations of peripheral neurons (i.e., sensory, autonomic, and somatic motor neurons) (Escurat et al., 1990; Gorham et al., 1990). It can assemble into filaments by itself or with the three neurofilament (NF) proteins NF-light, NF-medium, and NF-heavy (Parysek et al., 1991; Beaulieu et al., 1999). While the exact function of peripherin in neurons is unknown, recent evidence indicates it is a normal subunit of neurofilaments (together with the three NF proteins) in peripheral neurons (Yuan et al., 2012). In PMAs from control rats, peripherin was expressed in axons within the thicker preterminal nerve fibers but was not detectable or weakly expressed in axons within the finer nerve terminal fibers that were closer to the vascular muscle surface (Figure 2B). Labeling for peripherin increased in finer nerve fibers of PMAs from STZ-LI rats (Figure 2F). As the average width of the peripherin IR intercepts along the line profiles also increased in PMAs from STZ-LI rats, these findings are consistent with an increase in axonal content of neurofilaments causing an increase in axon diameter (see Holmgren et al., 2012).

The findings for β-tubulin III indicate that there was an increase in neural tissue within the perivascular nerve plexus. However, whether this effect of STZ-induced diabetes involves an increase in the number of axons within the nerve fibers was not resolved. Other studies have indicated that STZ-induced diabetes stimulates growth of sympathetic nerve terminals in the heart (Felten et al., 1982), corpus cavernosum (Morrison et al., 2007), and seminal vesicle (Morrison et al., 2006). In both corpus cavernosum and seminal vesicle, the TH-IR of the nerve fibers was increased (Morrison et al., 2006, 2007). In addition, STZ-induced diabetes increased the NPY content of both the corpus cavernosum and seminal vesicle (Morrison et al., 2009). We have previously reported increased TH-IR of the perivascular nerve fibers in PMAs from STZ-LI rats (Johansen et al., 2013) and the present study demonstrated an increase in NPY-IR. Therefore, it appears that the changes occurring in perivascular nerve plexus of PMAs are similar to those occurring in other sympathetically innervated tissues.

Current evidence indicates that sympathetic nerve terminals normally undergo continuous cycles of degeneration and regeneration (i.e., they are continuously being remodeled) and that this process may be exaggerated by diabetes (Schmidt, 2002). Furthermore, it has been suggested that axonal structural changes produced by diabetes are the result of initial nerve damage followed by abnormal regeneration (Schmidt, 2002). In the longer term, the loss of sympathetic axons that is believed to occur in diabetic humans may be explained by the eventual failure of damaged axons to regenerate (Sima, 2003; Yasuda et al., 2003).

Sympathetic neurons express insulin receptors raising the possibility that insulin has a direct trophic influence on these neurons (James et al., 1993; Karagiannis et al., 1997). Previously we reported that the low dose of insulin used in this study prevented the reduction in both perivascular nerve fiber density and neurovascular transmission observed in STZ-treated rats that did not receive insulin support (Johansen et al., 2013). These findings clearly suggest that these more substantive changes are caused by the loss of insulin signaling rather than hyperglycemia. While in the present study the protective actions of insulin were observed with a dose that greatly reduced blood glucose levels, we cannot exclude possibility that the changes to the perivascular sympathetic nerve plexus axons observed in STZ-LI rats are also produced by the reduced trophic influence of insulin on sympathetic neurons. Indeed, reduced trophic support from insulin (or insulin growth factor-1) has been suggested be the major determinant of structural changes to sympathetic neurons supplying the intestine of STZ-treated rats (Schmidt et al., 2003).

The changes in the perivascular nerve plexus of STZ-LI rats were not associated with changes in neurovascular function (Johansen et al., 2013). Indeed, in PMAs from STZ-treated rats that did not receive any insulin, it is not possible to firmly conclude that the decrease in neurovascular transmission is explained by the observed decrease in nerve fiber density, as vascular reactivity to exogenously applied α-adrenoceptor agonists was also decreased (Johansen et al., 2013). This indicates that the reduction in neurovascular transmission can be attributed, at least partially, to a postjunctional change. Rarely have the effects of changes to the perivascular vascular innervation on neurovascular transmission been studied. Importantly, recent studies using surgical lesions have demonstrated in rat tail artery that changes in nerve-evoked contractions were only detected when greater than 50% of the perivascular nerves were removed (Tripovic et al., 2013). Similarly, during reinnervation of denervated tail arteries, neurovascular function returned to control levels when the perivascular nerve density was less than control values (Tripovic et al., 2011). These findings suggest there can be substantial changes to the perivascular sympathetic innervation without measurable changes in nerve-evoked contractions.

To our knowledge other microscopic studies of sympathetic nerves supplying arterial vessels from rats with STZ-induced diabetes have not detected changes in nerve fiber structure, even with durations of diabetes in excess of 1 year (Schmidt et al., 1988). This suggests vasoconstrictor neurons supplying the planter vascular bed are particularly vulnerable to the effects of diabetes, as also appears to be the case in diabetic humans (Shore et al., 1994; Cacciatori et al., 1997). In humans, we can find no reports of changes to the perivascular nerve plexus of blood vessels produced by diabetes. However, long-term type 1 and type 2 diabetic patients with neuropathic pain in their feet had reductions in both norepinephrine release and the uptake of the sympathoneural imaging agent 6-[^18^F]fluorodopamine (imaged by positron emission tomography) in their feet (Tack et al., 2002). These findings suggest that long-term diabetes can produce partial sympathetic denervation of the feet. Importantly, none of the diabetic patients studied by Tack et al. (2002) displayed characteristic signs of diabetic sympathetic neuropathy (i.e., no orthostatic hypotension, normal blood pressure responses to the Valsalva maneuver, and normal increments in plasma norepinephrine levels during standing), consistent with the preferential vulnerability of sympathetic neurons supplying the feet to the neurotoxic effects of diabetes. This increased vulnerability may result from the long axon length of the vasoconstrictor neurons supplying the feet, as has been described for somatic sensory and motor neurons (Anand et al., 1996). Therefore, it is possible that the effects of STZ-induced diabetes on the sympathetic innervation of PMAs with 12 weeks of STZ-induced diabetes represent early changes that will lead to nerve fiber loss in the longer term.

The question remains whether similar changes to those we have described in STZ-treated rats also occur in humans with type 1 diabetes. While there appears to be no direct investigations of perivascular sympathetic nerves in human diabetics, there are reports of increased, rather than decreased, sympathetic vasomotor regulation in young type 1 diabetics (e.g., Dalla Pozza et al., 2007; Lucini et al., 2009). In addition, a number of studies have reported that reductions in parasympathetic control of the heart in type 1 diabetic patients precede impairment of sympathetic vasomotor regulation (e.g., Javorka et al., 2005; Dalla Pozza et al., 2007). These findings suggest vasoconstrictor sympathetic neurons are less vulnerable to the neurotoxic effects of Type 1 diabetes. In plantar skin of patients with type 1 diabetes for less than 3 years, there were increases in vasoactive intestinal peptide (VIP) IR nerve fibers surrounding sweat glands, indicating increased cholinergic sympathetic innervation (Properzi et al., 1993). With longer durations of diabetes, the numbers of VIP-IR fibers surrounding sweat glands were significantly reduced. A similar increase in the VIP-IR nerve fibers in the vicinity of sweat glands has been demonstrated in skin from rats with 12 weeks of STZ-induced diabetes (Karanth et al., 1990). These findings suggest that type 1 diabetes initially induces growth of cholinergic sympathetic neurons and that the changes seen at the 12 week time point in STZ-treated rats are similar to those observed in the initial stages of neuropathy in humans with type 1 diabetes. However, whether type 1 diabetes stimulates growth of perivascular noradrenergic sympathetic neurons at initial stages of type 1 diabetes in humans remains to be determined.

It is concluded that there are changes to the sympathetic innervation of the PMAs before there are changes in neurovascular function. It is possible that the changes observed in the present study are related to the remodeling and regrowth of sympathetic nerve endings damaged early in this model of diabetes, as has been suggested in the intestine (Schmidt, 2002). Furthermore, as these changes were not observed in STZ-HI rats, they might be induced by hyperglycemia-induced nerve damage and/or reduced trophic support from insulin. The findings suggest that changes in peripherin within the perivascular nerve plexus may provide an early indication of diabetes induced changes in perivascular nerves. In addition, they highlight the possibility that early interventions will be needed to prevent the onset of diabetes-induced changes in sympathetic neurovascular transmission.

## Author contributions

Niloufer J. Johansen, Tony Frugier, Billie Hunne, James A. Brock contributed to the conception and design of the research. Niloufer J. Johansen performed the experiments. Niloufer J. Johansen, Tony Frugier, Billie Hunne, James A. Brock analyzed the data. Niloufer J. Johansen and James A. Brock interpreted the results of the experiments. Niloufer J. Johansen and James A. Brock prepared figures and drafted the manuscript. Niloufer J. Johansen, Tony Frugier, Billie Hunne, and James A. Brock revised the manuscript and approved the final version.

### Conflict of interest statement

The authors declare that the research was conducted in the absence of any commercial or financial relationships that could be construed as a potential conflict of interest.

## Funding

National Health and Medical Research Council of Australia (ID 568850).
